# Design of 3D Additively Manufactured Hybrid Structures for Cranioplasty

**DOI:** 10.3390/ma14010181

**Published:** 2021-01-02

**Authors:** Roberto De Santis, Teresa Russo, Julietta V. Rau, Ida Papallo, Massimo Martorelli, Antonio Gloria

**Affiliations:** 1 Institute of Polymers, Composites and Biomaterials, National Research Council of Italy, V.le J.F. Kennedy 54–Mostra d’Oltremare Pad. 20, 80125 Naples, Italy; teresa.russo@cnr.it (T.R.); antonio.gloria@cnr.it (A.G.); 2 Istituto di Struttura della Materia, Consiglio Nazionale delle Ricerche (ISM-CNR), Via del Fosso del Cavaliere 100, 00133 Rome, Italy; giulietta.rau@ism.cnr.it; 3 Department of Analytical, Physical and Colloid Chemistry, Institute of Pharmacy, Sechenov First Moscow State Medical University, Trubetskaya 8, Build. 2, 119991 Moscow, Russia; 4 Department of Advanced Biomedical Sciences, University of Naples Federico II, 80131 Naples, Italy; ida.papallo@unina.it; 5 Department of Industrial Engineering, Fraunhofer JL IDEAS, University of Naples Federico II, P.le Tecchio 80, 80125 Naples, Italy; massimo.martorelli@unina.it

**Keywords:** reverse engineering, design for additive manufacturing, composite bone cement for cranioplasty, temperature profile analysis, finite element analysis

## Abstract

A wide range of materials has been considered to repair cranial defects. In the field of cranioplasty, poly(methyl methacrylate) (PMMA)-based bone cements and modifications through the inclusion of copper doped tricalcium phosphate (Cu-TCP) particles have been already investigated. On the other hand, aliphatic polyesters such as poly(ε-caprolactone) (PCL) and polylactic acid (PLA) have been frequently investigated to make scaffolds for cranial bone regeneration. Accordingly, the aim of the current research was to design and fabricate customized hybrid devices for the repair of large cranial defects integrating the reverse engineering approach with additive manufacturing, The hybrid device consisted of a 3D additive manufactured polyester porous structures infiltrated with PMMA/Cu-TCP (97.5/2.5 *w*/*w*) bone cement. Temperature profiles were first evaluated for 3D hybrid devices (PCL/PMMA, PLA/PMMA, PCL/PMMA/Cu-TCP and PLA/PMMA/Cu-TCP). Peak temperatures recorded for hybrid PCL/PMMA and PCL/PMMA/Cu-TCP were significantly lower than those found for the PLA-based ones. Virtual and physical models of customized devices for large cranial defect were developed to assess the feasibility of the proposed technical solutions. A theoretical analysis was preliminarily performed on the entire head model trying to simulate severe impact conditions for people with the customized hybrid device (PCL/PMMA/Cu-TCP) (i.e., a rigid sphere impacting the implant region of the head). Results from finite element analysis (FEA) provided information on the different components of the model.

## 1. Introduction

Cranioplasty is the surgical procedure for correcting cranial deformities or defects arising from skull fracture, deformities, cancer and infections. The repair or regeneration of cranial defects involves the use of biomaterials, and several approaches, based on polymers and composites, can be distinguished [1,2,3].

The in situ application of bone cement such as poly(methyl methacrylate) (PMMA) represents the most common approach. This cement consists of a solid powder phase made of PMMA and a liquid monomer; by mixing these two phases, a radical polymerization reaction occurs driven by benzoyl-peroxide and amines [4,5,6]. Such biomaterial is used for bone reconstruction from more than half a century, and the cranioplasty procedure consists of a single intra-operatively step [7], as the malleable paste is applied onto the cranial defect, and it is easily shaped around the contours of the patient’s skull defects [4,8]. Once polymerized, mechanical properties are in between those of cortical and spongy bone [9,10]. The PMMA skull prosthesis is commonly fixed through titanium screws and plates [11]. In order to prevent cranioplasty graft infections [4,12], gentamicin has been loaded into PMMA [13,14], but a reduction in mechanical properties has been shown [6]. The incorporation of silver [15] and gold [16] nanoparticles into PMMA bone cements has been suggested for antimicrobial and mechanical purposes. Recently, novel antibacterial agents, such as bioactive glass (BG) and copper doped tricalcium phosphate (Cu-TCP) particles, have been incorporated into surgical PMMA in order to prevent cranioplasty graft infections [17]. A low amount of Cu-TCP (i.e., 2.5 wt%) showed an efficient antibacterial effect while providing a mechanical reinforcement for the polymer matrix [17].

The in situ approach is considered convenient as it reduces the time from diagnosis to implantation, but heat and shrinkage due to the polymerization process as well as the release of unreacted monomers represent the main drawbacks. The most significant drawback of the in situ approach for large cranial defects based on PMMA is the heat developed as a consequence of the exothermic reaction. To overcome this issue, the ex vivo approach based on the molding strategy has been developed. The mold can be realized through a plaster impression previously taken with a gel or a wax over the skull defect, a silicone mold is then fabricated, and the PMMA skull prostheses is finally formed by pouring the cement into the mold [11,18].

Among the ex vivo approach, 3D scan of the cranial defect in conjunction with Additive Manufacturing (AM) technologies allow the manufacture of the customized mold into which the PMMA paste is poured and formed [19,20,21]. In recent years, AM has replaced the computer-aided design (CAD)/computer-aided manufacturing (CAM) milling method for manufacturing the mold replicating the skull defect starting from X-ray computed tomography (CT) [22]. However, photogrammetry and Laser scanners are gaining popularity in the field of clinical 3D imaging tools as they are less invasive than X-ray CT [23,24]. The process by which 3D skull scans are analyzed and converted into suiTable 3D virtual models is known as Reverse Engineering (RE) [1]. The combination of the RE approach with AM has allowed the direct fabrication of the skull defect and, hence, the manufacturing of the mold. Ink-Jet Printing (IJP), Fused Deposition Modeling (FDM), Laser Sintering (LS) and Stereolithography (SLA) represent the main AM technologies employed for the fabrication of a synthetic replica of the skull defect [1]. A silicone mold has been manufactured using the positive shape of the cranial defect previously fabricated through IJP processing a photopolymerizable resin [25]. Using a similar strategy, the positive shape of the cranial defect has been additively manufactured through SLA, and a composite skull prosthesis has been realized into an alginate mold [20,26]. The main advantage of SLA is the high accuracy in reproducing the solid 3D model [27]. A silicone mold has been manufactured from the positive shape of the cranial defect previously realized processing polyamide powders through LS [28]. Similarly, the mold has been fabricated around the positive shape of the acrylonitrile-butadiene-styrene cranial defect manufactured through FDM [4,8]. Instead, by using the FDM approach, an acrylonitrile-butadiene-styrene mold, directly reproducing the skull defect, has been produced, and paraffin oil has been spread over the mold walls to prevent the sticking of the PMMA prostheses [3].

The integrated approach combining 3D scanning, RE and AM [29,30,31] represents the most recent and advanced approach to directly fabricate skull prosthesis or scaffold. Biodegradable polymer-based scaffolds for cranial bone regeneration are commonly manufactured by FDM. With this technology, a continuous thermoplastic filament (e.g., polyesters and their copolymers) is deposited from the melt state, the scaffold porosity and pore dimension are determined by process and geometrical parameters such as the strand distance and the layer stacking sequence [32]. In the case of non-degradable devices (i.e., prosthetic approach), skull growth clearly adds an additional concern in cranioplasty; thus, biodegradable structures (i.e., scaffolds) for cranial bone tissue engineering are particularly important for pediatric patients [31]. Poly(ε-caprolactone) (PCL), polylactic acid (PLA) and poly(lactic-*co*-glycolic acid) (PLGA) represent the most common polyesters used to manufacture biodegradable scaffolds for cranial bone regeneration [31,32,33,34,35]. PLGA scaffolds, manufactured by FDM and implanted in the parietal skull defect, have shown evident angiogenesis within three weeks of in vivo observations in a mice model [36]. PCL is a degradable polyester easy to process using FDM as its melting temperature is low (60 °C) compared to the other polyesters; furthermore, its mechanical properties are similar to dense spongy bone [36,37]. PCL has been processed by FDM, and scaffolds for cranial bone regeneration of critical-size defects have been investigated in the rabbit [38] and human [39] models.

The design of innovative 3D lattices and porous devices clearly spans from industrial to biomedical applications. Many progresses in 3D bioprinting of hydrogel-based biomaterials have also been recently discussed from the tissue engineering perspective. In this case, differently from the prosthetic approach, the attention has been focused on hydrogel-based bioprinted scaffolds to develop functional tissues through the use of advanced fabrication methods covering multiple-dispenser, coaxial and hybrid 3D printing processes [40].

Further progresses in the field of additive manufacturing were also related to the introduction of four-dimensional (4D) printing technology to develop tunable continuous-stable metamaterials with reversible thermo-mechanical memory operations [41]. Experimental and numerical tests were carried out to analyze a 3D printed tunable reversible mechanical metamaterial unit with bi-stable memory operations. Cold and hot programming were suitably combined, and the potential to mimic electronic memory devices as well as to design surface adaptive structures was demonstrated. In the biomedical field, the possibility to develop advanced devices (i.e., self-deployable stents) was stressed [41]. Several combinations of hard and soft components were adopted to fabricate dual-material lattice-based meta-structures through 4D printing FDM technology. As an example, an approach towards 4D printing tunable meta-sandwiches was already reported for applications concerning reversible energy absorption [42].

Even though many progresses have been made in the design of lattice structures, 3D printed scaffolds for tissue regeneration and advanced prosthesis [30,31,36,39,40,43,44,45,46,47], the aim of the current investigation was to design and fabricate customized hybrid devices as a further alternative for the repair of large cranial defects, integrating the reverse engineering approach with additive manufacturing. The hybrid device consisted of a 3D additive manufactured polyester porous structures infiltrated with PMMA/Cu-TCP (97.5/2.5 *w*/*w*) bone cement. Temperature profiles were first evaluated during setting also in the case of the hybrid devices. In addition, to the best of the authors’ knowledge, none of the currently existing human head finite element models consider the impact-related features in the case of people using prosthetic devices for a large cranial defect. For this reason, a theoretical analysis was preliminarily performed on the entire head model trying to simulate a people with the customized hybrid device (PCL/PMMA/Cu-TCP) in severe impact conditions (i.e., a rigid sphere impacting the implant zone of the head).

## 2. Materials and Methods

### 2.1. Modified Bone Cement

Copper-doped tricalcium phosphate (Cu-TCP) particles were employed to modify a PMMA-based cement (Palacos, Heraeus, Wehrheim, Germany). Specifically, the precipitation technique was used to obtain Cu^2+^-substituted TCP as previously described [17]. In brief, 0.5 mol/L solution of Cu(NO_3_)_2_ was mixed with 0.5 mol/L solution of Ca(NO_3_)_2_, and 0.5 mol/L (NH_4_)_2_HPO_4_ solution was added. Ammonia solution was added to the solution for keeping the pH at 6.5–6.9. After 30 min, the formed precipitate was filtered and washed using distilled water.

The precipitate was then dried at 80 °C and calcined at 900 °C forming the whitlockite structure. PMMA cement was modified according to a previously reported procedure [17]. Cu-TCP particles were dispersed in the solid PMMA phase using ultrasonic dispersion. The liquid phase was then added and hand-mixed to the solid phase. Benefiting from the previous results, a specific formulation was considered for PMMA/Cu-TCP (97.5/2.5 *w*/*w*) [17].

### 2.2. Design and Fabrication of 3D Additive Manufactured Porous Structures

Three-dimensional porous structures with different lay-down patterns (0°/90° and 0°/45°) were fabricated by an additive manufacturing technique based on injection/extrusion methods (i.e., Fused Deposition Modeling), using a commercially available 3D printer (Creality3D Ender-3 PRO) and two different aliphatic polyesters (poly(ε-caprolactone)—PCL and polylactic acid—PLA). In brief, PCL (Facilan^TM^ PCL 100—density: 1.1 g/cm^3^, melting point: 58–60 °C) or PLA (FILOALFA—density: 1.24 g/cm^3^, melting point: 135 °C) filaments (1.75 mm in diameter) were heated, and the 3D polymeric structures were manufactured by injecting/extruding the material through a nozzle (inner diameter of 500 µm).

The filaments were deposited along specific directions between two successive layers according to the adopted lay-down pattern. The filament distance (i.e., center-to-center distance) and layer thickness were set to 1000 and 400 µm, respectively. A printing speed of 15 mm/s was used.

### 2.3. Temperature Profile Evaluation

**Figure 1:**
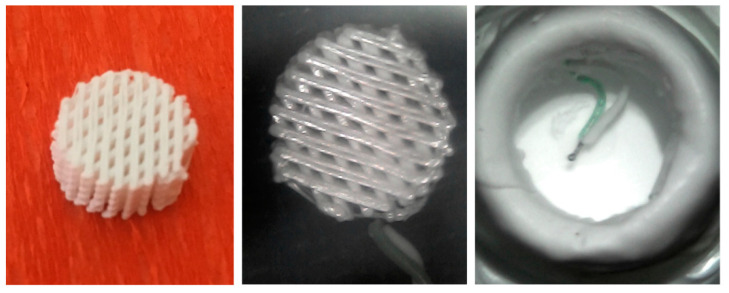
Typical image of a 3D printed porous structure (diameter of 10 mm, height of about 5 mm) (**left**); 3D porous structure incorporating a k-type thermocouple (**center**); hollow cylindrical Teflon mold equipped with k-type thermocouple (**right**).

The effect of the employed thermoplastic polyesters on the exothermal reaction occurring during cement setting was investigated by recording temperature profiles. Specifically, cylindrical 3D printed PCL and PLA structures (diameter of 10 mm, height of about 5 mm) with a fully interconnected pore network (porosity of 50%) (Figure 1—left). Each 3D structure was equipped with a disposable k-type thermocouple (Figure 1—center).

Hollow cylindrical Teflon molds (inner diameter of 12 mm and height of 20 mm) were employed and equipped with k-type thermocouples (Figure 1—right), The k-type thermocouples connected to the National Instruments DAC interface and the LabView system were employed for temperature measurements. The mold was positioned onto a thermoblock system allowing the control of the base line temperature of the Teflon mold at 37 °C.

PMMA and PMMA/Cu-TCP (97.5/2.5 *w*/*w*) cement pastes were poured into the Teflon molds equipped with the thermocouple (Figure 1—right). Heat was released during setting, and temperature was recorded over time for 1000 s. Five specimens were considered for each kind of bone cement.

PCL/PMMA and PLA/PMMA hybrid specimens were obtained by placing each 3D cylindrical porous structure into the Teflon mold and then pouring the bone cement pastes. A Teflon piston was manually used to gently press the cement into the mold for promoting the infiltration of PMMA bone cement into the pore network of the structure. Five specimens were considered for each kind of structure. The measured peak temperatures were reported as mean value ± standard deviation. Statistical analysis was performed by analysis of variance (ANOVA). Statistical significance was set at *p* < 0.05.

### 2.4. Design and Fabrication of 3D Customized Hybrid Devices for Large Cranial Defects

**Figure 2:**
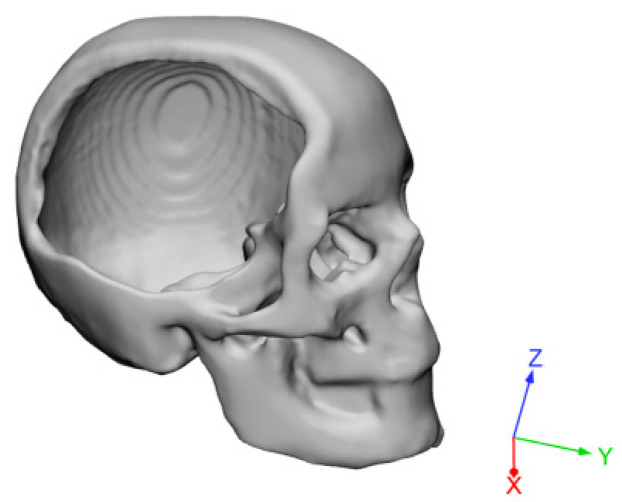
Three-dimensional reconstruction of a skull with a large cranial defect. The images were analyzed starting from a previous 3D scanning process [1].

Previous results obtained from image capture and analysis techniques were used to generate a 3D virtual model of a skull with a large cranial defect (Figure 2).

**Figure 3:**
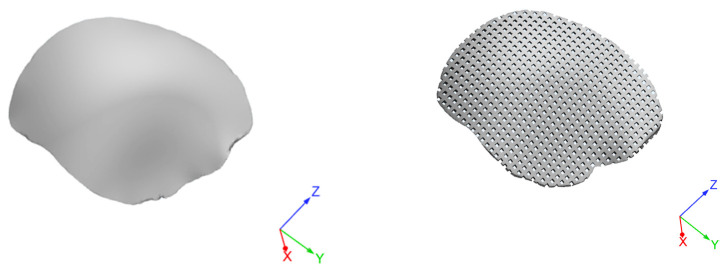
Three-dimensional geometrical models of a customized device for large cranial defect: non-porous model (**left**); model with interconnected pore network (**right**).

A SolidWorks^®^2017 (Dassault Systemes, Paris, France) computer-aided design (CAD) system was employed to create 3D customized porous models of devices for cranioplasty. Starting from a non-porous geometrical model, a porous model was then created while maintaining constant a porosity of about 50% to allow the cement infiltration (Figure 3).

**Figure 4:**
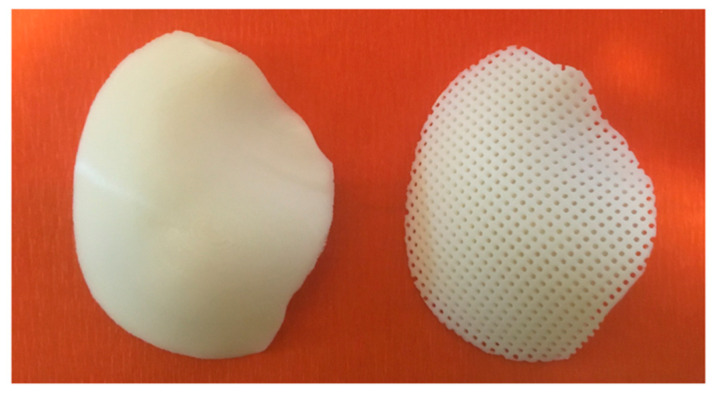
Three-dimensional additive manufactured devices for large cranial defect: non-porous model (**left**); model with interconnected pore network (**right**).

Customized devices consisting of PCL or PLA were also additive manufactured by FDM. As an example, Figure 4 reports an image of two models of customized PCL devices for large cranial defect.

**Figure 5:**
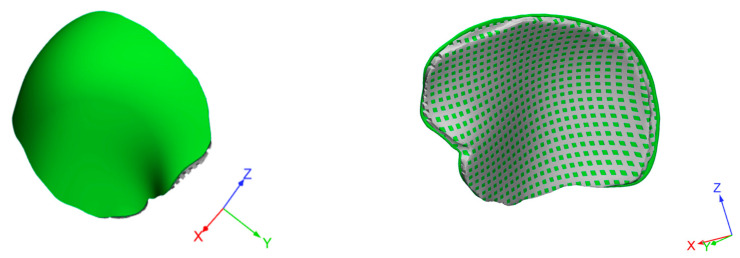
Three-dimensional geometrical models of a customized hybrid device for large cranial defect: top view evidencing the external cement layer (**left**); bottom view highlighting the cement infiltration in the fully interconnected pore network (**right**). Green color was chosen to identify the cement component of the hybrid device.

Three-dimensional hybrid devices were physically obtained through cement infiltration (PMMA/Cu-TCP 97.5/2.5 *w*/*w*) in the fully interconnected pore network of the 3D additive manufactured structures. An external upper layer of bone cement was properly realized. Thus, the additive manufactured hybrid structures were also geometrically modeled (Figure 5).

### 2.5. Theoretical Impact Analysis

The geometry of an adult human head was obtained from a previous study [1]. Benefiting from early studies [48,49], the main anatomical features were modeled (i.e., skull, falx, tentorium, subarachnoid space, scalp, cerebrum, cerebellum, brainstem), also taking into account their properties. Scalp (16.7 MPa, 0.42), cerebral spinal fluid (CSF) (0.012 MPa, 0.49), tentorium (31.5 MPa, 0.45) and falx (31.5 MPa, 0.45) were assumed to be isotropic, homogeneous and elastic. The values reported in the brackets are the elastic modulus and Poisson’s ratio for the different element of the model.

Brain was assumed to be viscoelastic considering the shear relaxation behavior with G_0_ (1.66 kPa) and G_∞_ (0.93 kPa) as the short-term and long-term shear modulus, respectively, and b (16.95 s^−^^1^) as the decay coefficient [49,50]. A three-layer shell was employed to model the skull, the aim being to represent the external table, the middle porous layer and the inner table of human cranial bone. With regard to the cortical bone (i.e., inner and outer table) an elastic modulus of 15,000 MPa and a Poisson’s ratio of 0.22 were considered, whereas values of 1000 MPa and 0.24 were used for the cancellous bone [49].

The customized additive manufactured hybrid device consisting of PCL (380 MPa, 0.40) and the infiltrated PMMA/Cu-TCP (97.5/2.5 *w*/*w*) bone cement (3200 MPa, 0.30) were the further components of the finite element analysis (FEA) model. Concerning the cement, the external upper layer and the part infiltrated into the interconnected pore network were modeled as a single block component.

The entire head model with the hybrid device for the large cranial defect was imported into HyperMesh^®^ (HyperWorks^®^, Altair Engineering Inc., Troy, MI, USA).

**Figure 6:**
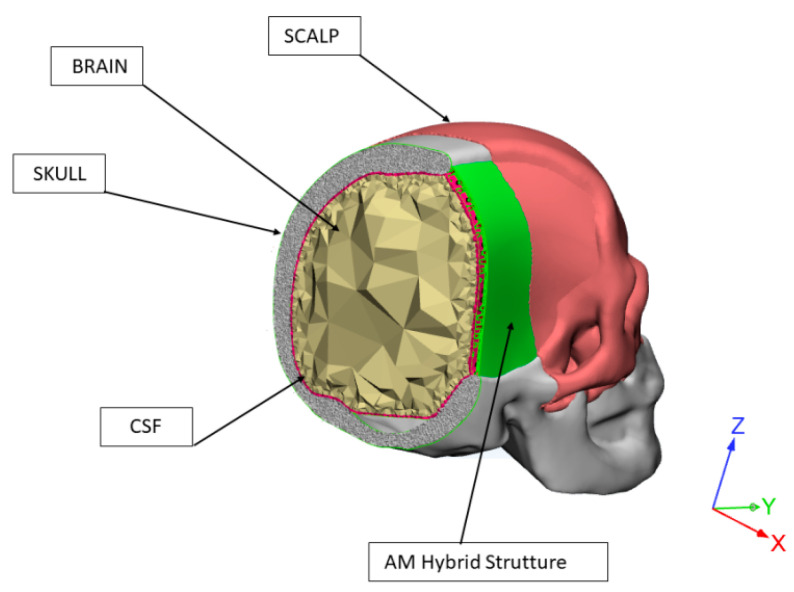
A simplified image reporting some components of the finite element analysis (FEA) model.

A 3D mesh was properly generated, adequate mesh size and mesh refinement techniques were used. Different contact types were considered for the several parts of the head. Surface-to-surface contacts were used between the scalp and skull as well as between the scalp and the external cement layer of the hybrid device. Tied surface-to-surface contacts were considered in the case of the other elements. Impact analysis was performed using Altair Radioss^TM^ (Altair Engineering Inc., Troy, MI, USA), which is a structural analysis solver for highly non-linear problems under dynamic loadings. To simulate severe impact conditions, the head model was impacted on the region of the customized hybrid device by a 50 mm diameter rigid sphere (elastic modulus of 210,000 MPa, Poisson’s ratio of 0.30, mass of 0.463 kg) moving at a speed of 7 m/s along the *x*-axis (opposite verse) (Figure 6). The impactor was in contact with the scalp surface. A friction coefficient of 0.3 was considered between the scalp and the impactor. The whole head was properly constrained.

The von Mises stress distributions were evaluated for the different components of the model.

## 3. Results and Discussion

**Figure 7:**
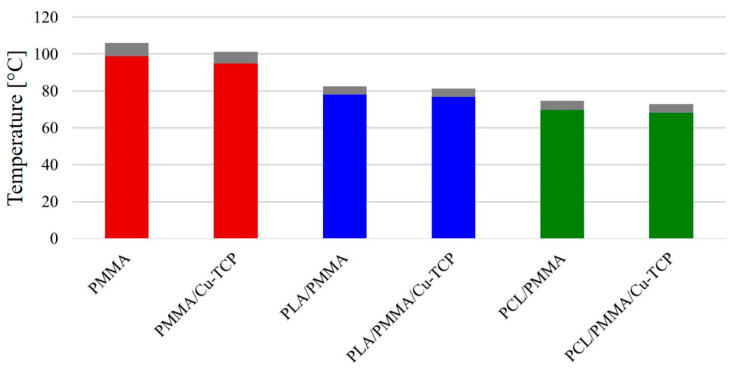
Temperature peaks measured during setting in the case of plain bone cements (PMMA, PMMA/Cu-TCP) and hybrid structures consisting of 3D polylactic acid (PLA) or poly(ε-caprolactone) (PCL) networks infiltrated with cement. Statistical analysis was performed by analysis of variance (ANOVA). Statistical significance was set at *p* < 0.05.

Figure 7 reports temperature peaks measured during setting of PMMA and PMMA/Cu-TCP bone cement. Mean peak temperature of plain PMMA cement (98.9 ± 7.0 °C) was not significantly different than that of PMMA/Cu-TCP (95.8 ± 6.3 °C). Irrespective of the type of bone cement, mean peak temperature of the cement-infiltrated PLA structure (PLA/PMMA) was significantly lower than that of the plain cements (*p* < 0.05). Furthermore, no statistically significant differences were found between PLA/PMMA (78.2 ± 4.5 °C) and PLA/PMMA/Cu-TCP (77.0 ± 4.9 °C).

Furthermore, peak temperatures recorded for PCL structures infiltrated with bone cement (*p* < 0.05) were significantly lower than those found for the PLA ones. However, in terms of mean peak temperature, no significant differences were observed between PCL/PMMA (69.5 ± 5.1 °C) and PCL/PMMA/Cu-TCP (67.8 ± 4.9 °C).

Figure 7 shows that peak temperature levels occurring in the setting of PMMA and PMMA/Cu-TCP cements are higher than 90 °C. As PMMA cements are used in conjunction with porous polyester structures, a significant reduction in the temperature peak level can be observed.

This temperature reduction is partially due to the amount of the acrylic resin. In fact, PLA and PCL structures have a fully interconnected porosity of 50%; therefore, the polyester structures infiltrated with PMMA presents a volume amount of acrylic resin which is equal to half of that of plain PMMA specimens. Hence, the heat developed through the polymerization phase of plain PMMA specimen is higher than that occurring in PLA/PMMA and PCL/PMMA. The further reduction in peak temperature observed for PCL/PMMA and PCL/PMMA/Cu-TCP can be ascribed to the peculiar thermal feature of this aliphatic polyester. PCL is a thermoplastic polymer with a melting temperature of about 60 °C, also showing a thermal regulating capability as PMMA polymerization is concerned [51].

**Figure 8:**
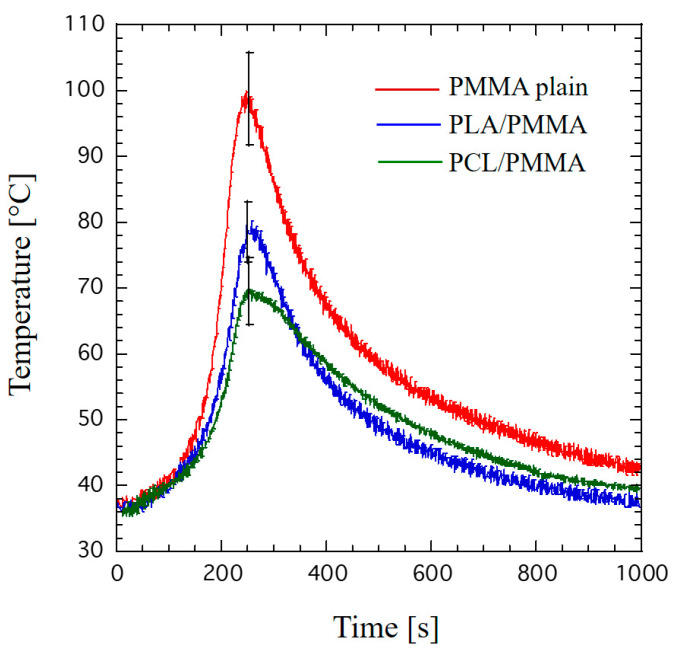
An example of temperature profiles: plain bone cement, PLA and PCL structures infiltrated with PMMA, evidencing some differences during the cooling phase.

Figure 8 reports the temperature profiles obtained for the plain bone cement as well as in the case of PLA and PCL structure infiltrated with PMMA. Although the PLA structures infiltrated with PMMA showed a temperature peak significantly lower (*p* < 0.05) than the plain bone cement, a similar temperature profile was observed. Indeed, looking at Figure 8, a different temperature profile can be distinguished for PCL structures infiltrated with PMMA during the cooling phase.

The endothermic process, which is due to the phase change and should mainly occur at the PCL fiber surface, should compensate the exothermal polymerization reaction. The effect is a further significant reduction in peak temperature (Figure 7). The heat absorbed by the PCL phase in the melting stage is then released in the cooling stage of PCL/PMMA (Figure 8). Even though the employed PLA has a melting point of 135 °C (i.e., higher than that of PCL), it has a glass transition temperature of about 55–60 °C, and it is well known that the glass–rubber transition occurs as the temperature is increased. However, as a consequence, the cooling profile of PCL/PMMA is more spread than that of PLA/PMMA over the observed time period.

Contextually, an integrated approach involving the combination of RE and AM was considered to design virtual and physical models of customized devices for large cranial defects.

**Figure 9:**
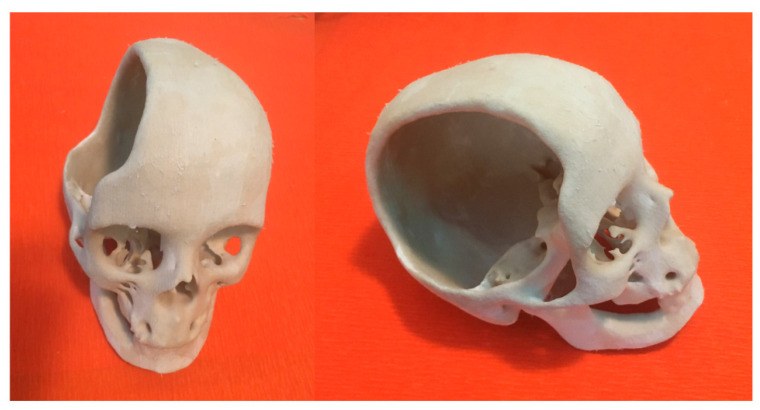
Images of the 3D physical model of a skull with a large cranial defect, which was previously fabricated by inkjet printing, starting from image capture and analysis techniques [1]: top-front view (**left**) and lateral view (**right**).

A skull model containing a large cranial defect was previously 3D printed by integrating the RE and AM approaches [1]. An inkjet printer was used to additively manufacture the 3D physical model of the skull (Figure 9).

Virtual models of the skull with the large defect and the additive manufactured prosthetic device were created. Starting from the shape and size of the large cranial defect, the geometry of the porous device was properly designed to be fitted in the large defect cavity.

**Figure 10:**
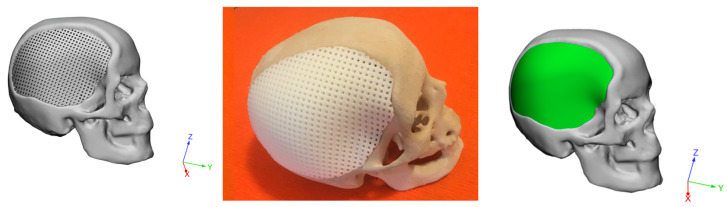
Feasibility of the reported technical solutions: images of virtual (**left**) and physical (**center**) models of skull with 3D additive manufactured PCL porous structure for large cranial defect 3D porous structure; image of virtual model of skull with 3D hybrid device (3D PCL porous structure infiltrated with PMMA/Cu-TCP 97.5/2.5 bone cement) for large cranial defect (**right**).

The feasibility of the proposed technical solutions was preliminarily assessed through virtual and physical models (Figure 10), evidencing the potential to adapt and conform the device to the contours of the large cranial defect.

The mechanical and morphological properties of different kinds of bone cements [10,16,17] as well as of 3D printed PCL and PLA structures [43,44,47,52] were already investigated. The consistency between real and theoretical values of the fiber diameter and pore size was assessed through scanning electron microscopy and micro-computed tomography. In particular, the effects of the designed lay-down patterns (i.e., sequences of fiber stacking), as well as of the pore shape and size, on the mechanical (e.g., modulus, maximum stress), mass transport and biological performances of 3D additive manufactured PCL structures were reported and also discussed from the tissue engineering perspective [43,44,47].

It has been frequently reported that traumatic brain injury is generally related to road traffic accidents, falls, sports, bullets, explosions and other kinds of external forces [49,53]. Road traffic accidents cause mortalities and most serious head injuries [49]. Moreover, measurements of the intracerebral local field potential are generally performed to monitor the brain activity and to further understand the information flow across the neural networks. In this context, a nano/micro-scale porous surface topology was also considered to develop enhanced neural electrodes able to measure higher amplitudes with lower noise levels, if compared to the conventional brain electrodes [54].

However, head impact injury represents a critical societal challenge as it may be considered the leading cause of disability and death [49].

Accordingly, it is fundamental to understand the injury mechanisms of the head as a consequence of trauma events through a biomechanical analysis.

In sum, this should be important for healthy people and especially for people using prosthetic devices for large cranial defects. For this reason, in recent years, most of researchers’ attention has been focused on the development of protection strategies and FEA models for a better understanding of the biomechanical response of the entire head in severe impact conditions.

In addition to traumatic brain injury, severe head injuries such as skull fracture obviously need further analyses. In this scenario, some scientific works have already reported experimental and theoretical studies on the skull and brain responses in several impact conditions (e.g., free falls, blunt and ballistic impacts), in many cases involving different kinds of impactors and tests on cadavers [48,49,55,56,57,58,59,60]. An advanced human head finite element model was also developed using a multiblock approach to predict skull response and brain pressure [49].

To the best of the authors’ knowledge, none of the above mentioned human head models considered the impact-related features in the case of people using prosthetic devices for a large cranial defect.

Benefiting from previous models and results [48,49,55,56,57,58,59,60], in the current research, an entire head model was developed also taking into account the presence of the designed hybrid device for a large cranial defect as well as a direct impact on the implant region of the head in severe conditions.

**Figure 11:**
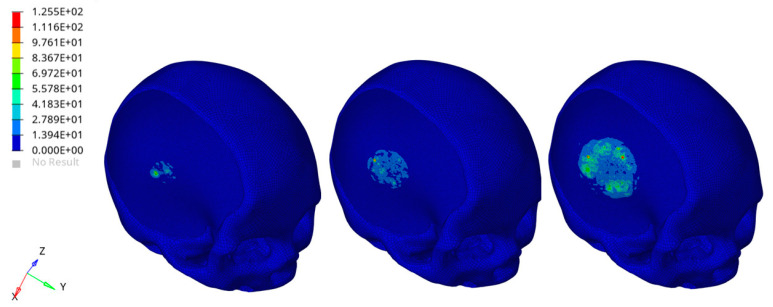
Biomechanical response as a result of the rigid sphere impacting the implant region of the head: von Mises stress (MPa) distributions for the external cement layer of the hybrid device at three different times after the initial impact (**left**). The figure is a guide for the eye to see the effect of the impact for the selected component. The color scale was chosen to allow for comparison among the models ad different times.

**Figure 12:**
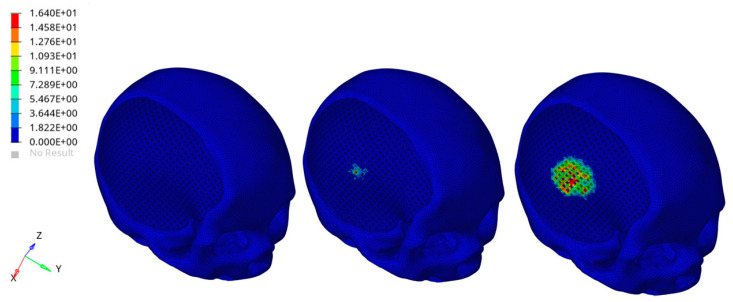
Biomechanical response as a result of the rigid sphere impacting the implant zone region of the head: von Mises stress (MPa) distributions for the 3D PCL structure underneath the external cement layer and infiltrated with the bone cement, at three different times after the initial impact (**left**). Bone cement was removed to visualize the effect of the impact for the selected component. The color scale was chosen to allow for comparison among the models at different times.

A preliminary FEA provided information in terms of von Mises stress distributions in the different components of the model (Figure 11 and Figure 12).

In Figure 11 and Figure 12, the color scale was chosen to allow for comparison among the models at different times. Stress results on the external cement layer was of the same order of magnitude of those reported for the skull obtained using a rigid hemispherical anvil (radius of 48 mm, density of 2700 kg/m^3^, elastic modulus of 70,000 MPa, Poisson’s ratio of 0.33, mass of 1.234 kg) and speeds of 7.6, 7.3 and 7.1 m/s toward the head [49]. A specific number of elements clearly failed in the external cement layer of the hybrid device as a consequence of the impact of the rigid sphere (Figure 11), which represents a severe impact condition. However, the obtained FEA results also suggested that the impact did not significantly alter the mechanical stability of the 3D PCL structure infiltrated with the bone cement and underneath the external cement layer (Figure 12). The presence of the PCL porous structures embedded in the cement would also create a toughening effect, however, increasing the ability of the hybrid device to absorb energy and deform before failure.

Moreover, a brief estimation of the minimum pressure for CSF and von Mises stress for the brain was also made. Even though the direct impact mainly caused the failure of some elements of the external cement layer, the analysis provided values of CSF minimum pressure and brain von Mises stress which should not lead to subdural hematomas (SDH) and diffuse axonal injuries (DAI), also considering the results computed with previous models as well as the corresponding injury risk curves for healthy people [61].

Although the current research provided an integrated approach to design 3D additively manufactured hybrid structures for large cranial defects, several limitations concerning the preliminary theoretical analysis need to be summarized: (i) lack of validation of the model-predicted brain motion against the results already reported in the literature as well as of the inclusion of brain motion-related physics (e.g., bridging veins) [62,63,64]; (ii) head–neck complex was not considered, even if neck is generally not taken into account when the impact time would be too short for it to affect the kinematic response of the head [48,49]; (iii) physical features of the white matter (e.g., fiber orientation anisotropy) were missing.

Furthermore, it is worth remembering that as the development of devices for large cranial defects is among the most investigated and controversial topics in cranioplasty, contradictory opinions still remain about clinical procedures, materials and modeling features (e.g., elastic or viscoelastic behavior and material parameters for some components).

For this reason, the present study may be also considered as a first step of a future research in which a complex model with more physical features will be analyzed.

## 4. Conclusions

Despite the limitations of the current research, the following conclusions were drawn:

1. Peak temperatures measured for hybrid PCL/PMMA (69.5 ± 5.1 °C) and PCL/PMMA/Cu-TCP (67.8 ± 4.9 °C) were lower than those found for the PLA-based ones.
2. An integrated design strategy was employed to develop 3D hybrid devices for large cranial defects, involving RE, AM and a modified PMMA bone cement (PMMA/Cu-TCP 97.5/2.5 *w*/*w*). The feasibility of the proposed technical solutions was validated through virtual and physical models.
3. A theoretical impact analysis was preliminarily carried out on the entire head model. Severe conditions were simulated considering a rigid sphere impacting the implant zone of the head for people with the customized PCL/PMMA/Cu-TCP device. FEA results suggested that the impact caused the failure of some elements of the external cement layer, without significantly altering the mechanical of the underneath PCL structure infiltrated with the cement.

However, even though mechanical and morphological analyses were already performed on the 3D additive manufactured structures, strong limitations are clearly related to FEA, which can make an overall conclusion about the designed 3D hybrid devices surely difficult as further experimental tests must be carried out (e.g., several impact conditions, cadavers, analysis of the 3D network-cement interface, in vivo studies) and compared to the results obtained from simulations. The achieved findings should probably help to improve predictions of the impact of the proposed hybrid devices in cranioplasty research as well as in clinical practice. In sum, the current research can contribute to provide a further insight into the development of alternative devices for the repair of large cranial defects and may be also considered as the first step of a future complex research, aiming at the evaluation of the in vitro and in vivo performances of such devices.

## Data Availability

Data is contained within the article.
